# Two ways to complex karyotype in MDS—the role of del(5q) and *TP53*

**DOI:** 10.1038/s41408-025-01305-w

**Published:** 2025-05-19

**Authors:** Sandra Huber, Stephan Hutter, Constance Baer, Manja Meggendorfer, Gregor Hoermann, Wolfgang Kern, Torsten Haferlach, Claudia Haferlach

**Affiliations:** MLL Munich Leukemia Laboratory, Munich, Germany

**Keywords:** Cancer genetics, Myelodysplastic syndrome, Cytogenetics, Genetics research

Dear Editor,

Myelodysplastic neoplasms (MDS) are defined as clonal hematopoietic stem cell neoplasms characterized by cytopenias and morphological dysplasia, and ineffective hematopoiesis with an increased risk of leukemic transformation [1]. In addition, MDS are very heterogeneous with respect to their underlying genetic landscape encompassing also cytogenetic abnormalities [2]. In this regard, deletion (del) 5q is a common aberration in MDS and detected in around 10–20% of MDS patients with a higher frequency in *TP53-*mutated than wild-type cases (55% vs. 15% [3]). The 5th edition of the WHO classification (WHO-HAEM5) defined a category of “MDS with low blasts and 5q deletion” if at most one additional cytogenetic aberration other than monosomy 7 or 7q deletion is present and no biallelic *TP53* inactivation is found [1]. Non-therapy related cases of this entity are generally associated with a favorable prognosis unless associated with *TP53* alterations, which can be detected in 10–20% of cases (most commonly single-hit) [4]. Furthermore, del(5q) can also occur in other MDS entities, most typically in the context of complex karyotypes (CK; defined as ≥3 cytogenetic abnormalities) [5]. MDS with CK are associated with an unfavorable outcome, harbor additional cytogenetic abnormalities such as monosomy 7 or del(7q) and del(17p), and frequently show *TP53* alterations, which are multi-hit in 80–90% of cases [6]. Here we used genetic hierarchy analysis to clarify whether MDS with CK, including del(5q), arises from MDS cases in which isolated del(5q) is the primary abnormality.

The cohort comprised 729 MDS patients harboring a del(5q) (median age: 76 years [32–98]; female: 58%) whose diagnoses were established following WHO-HAEM4R [7] (sample collection: 2006–2023; Supplementary Table S1). All samples were analyzed by cytomorphology, cytogenetics, FISH, and a targeted NGS panel (Supplementary Methods). We analyzed the clonal composition at initial diagnoses. To make cytogenetic clone size estimates directly comparable to variant allele frequencies (VAFs) of mutations, cell counts of cytogenetic abnormalities (CA) determined by interphase FISH were converted to a VAF-like metric (CA-VAF) by halving the percentage of aberrant cells. (CA-)VAF estimates were used to evaluate clonal hierarchy of *TP53* and del(5q) using a VAF difference cut-off of ≥5% (Supplementary Methods). Details on survival analysis and statistics are provided in the Supplement. All patients gave their written informed consent for genetic analyses and to the use of laboratory results and clinical data for research purposes according to the Declaration of Helsinki. The study was further approved by the laboratory’s institutional review board.

Of all del(5q) patients, 42% (308/729) had an isolated del(5q) (MDS-iso5q; median age: 77 years; female: 72%), 50% (365/729) had CK (median age: 74 years; female: 46%) and 8% (56/729) neither fulfilled criteria for MDS-iso5q nor CK (Supplementary Table S1).

Both age (median age: 77 vs. 74 years; *p* = 0.001) and sex ratio (female: 72% vs. 46%; *p* < 0.001) differed significantly between MDS-iso5q and MDS-CK. Of note, 68% (209/308) of MDS-iso5q fell into WHO entity MDS with low blasts and del(5q) (Supplemetary Table S1), showing a female ratio of 74%, which is in line with published data [4]. The most frequently co-occurring somatic mutation in cases with del(5q) was *TP53*, detected in 54% (397/729) of cases (Fig. 1A, Supplementary Fig. S1), out of which *TP53* was the sole mutated gene in 63% (251/397). *TP53* mutations were more frequent in MDS-CK than in MDS-iso5q (87% vs. 20%; *p* < 0.001) and were also more commonly multi-hit in MDS-CK compared to MDS-iso5q (81% vs. 27% of *TP53* altered cases; *p* < 0.001) (Fig. 1B). While MDS-CK was dominated by *TP53* regarding mutations, in MDS-iso5q mutations were more evenly distributed among myeloid genes (Supplementary Fig. S1B). Notably, 91% (259/286) of MDS with biallelic *TP53* inactivation (MDS-bi*TP53*, according to WHO) showed CK (Supplementary Table S1). Thus, our results are comparable to other studies highlighting the strong association between *TP53* mutations, in particular multi-hit, and complex karyotypes [8, 9].

**Fig. 1:**
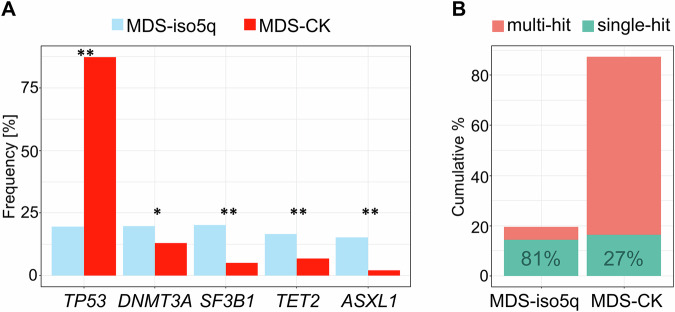
Co-mutations of MDS with del(5q). **A** The five most frequent co-mutations in MDS with del(5q) separated by entity (MDS-iso5q: blue; MDS-CK: red); *p < 0.05; **p < 0.001; CK complex karyotype. **B** Cumulative percentage of multi- and single-hit *TP53* altered cases separated by entity.

Next, we analyzed the clonal hierarchy of *TP53* mutations and del(5q) in 379 patients were FISH estimates for CA-VAF were available. The median CA-VAF of del(5q) was comparable in MDS-iso5q (*n* = 60) and MDS-CK (*n* = 319) (29% vs. 25%; *p* = 0.49), while the VAF of *TP53* mutations was lower in MDS-iso5q than in MDS-CK (19% vs. 32%; *p* < 0.001) (Fig. 2A). In this line, in MDS-iso5q the median CA-VAF of del(5q) was higher than the VAF of *TP53* mutations (29% vs. 19%; *p* = 0.006), while in CK cases the median CA-VAF of del(5q) was lower than the VAF of *TP53* mutations (25% vs. 32%; *p* < 0.001) (Fig. 2A). Congruently, the primary event (determined by VAF differences ≥5%) was different between entities (*p* < 0.001): in MDS-iso5q it was del(5q) in 48% (29/60) of cases compared to *TP53* mutations in 20% (12/60) of cases. In MDS-CK the primary event was del(5q) in 6% (20/319) of cases and *TP53* mutations in 46% (148/319) of cases (Fig. 2B). This pattern also held for more stringent definitions of the primary event (Supplementary Results; Supplementary Fig. S2). Overall, hierarchy analysis showed that most MDS-iso5q patients harbored del(5q) within the ancestral clone while *TP53* mutations where ancestral in a minority of cases. The fact that del(5q) is not universally an ancestral event was also shown in a previous study [10]. On the other hand, our data showed that in MDS-CK the ancestral clone encompassed *TP53* mutations in the majority of cases indicating that CK are more likely to arise from clones with a *TP53* mutation as the primary abnormality.

**Fig. 2:**
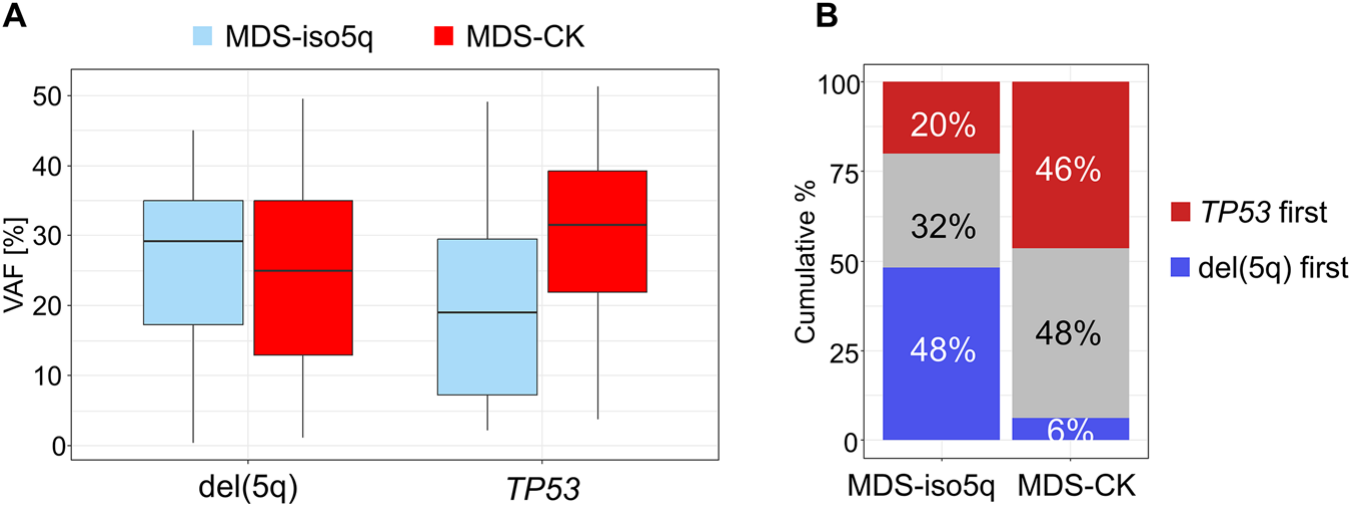
Clonal hierarchy analysis. **A** Boxplot of variant allele frequencies (VAF) of del(5q) (i.e., CA-VAF) and *TP53* mutations separated by entity (MDS-iso5q: blue; MDS-CK: red). CK complex karyotype. **B** Cumulative percentage of *TP53* mutations or del(5q) as primary event separated by entity. gray: hierarchy not determinable due to VAF differences <5%.

For a subset of patients data on karyotype and *TP53* status were available from different time points (median follow-up time: 2.8 years). Out of 84 patients initially diagnosed with MDS-iso5q 4% (3/84) presented with normal karyotype following transplantation or therapy with lenalidomide, 4% (3/84) showed clonal evolution (acquisition of one additional cytogenetic alteration), 24% (20/84) developed CK (acquisition of two or more additional cytogenetic alterations; median time to progression: 2.8 years [0.4–7.3]; 4.0 years [0.8–7.3] with lenalidomide treatment and 1.1 years [0.5–5.3] without (*p* = 0.08)) and 69% (58/84) showed a stable karyotype (Supplementary Fig. S3A). Disease progression was not a common event in MDS-iso5q patients concordant with other studies [4, 11]. *TP53* alterations were significantly more common at final follow-up in patients progressing to CK (13/20; 65%) than in those retaining a stable karyotype (22/58; 38%; *p* = 0.04), as were multi-hit *TP53* alterations which were found in 50% (10/20) of CK acquiring patients and only in 19% (11/58) of stable MDS-iso5q patients (*p* = 0.02) (Supplementary Fig. S3B, C; Supplementary Results). As also shown by others, *TP53* mutation status has prognostic impact in MDS patients with del(5q), and *TP53* multi-hit alterations are predictive of an increased risk of leukemic transformation [4, 8]. Comparable to the study of Montoro et al. [4], *TP53* allelic state significantly impacted overall survival in MDS-iso5q. However, this was not observed for MDS-CK (Supplementary Fig. S4). In line with previous studies [4, 12] *TP53* mutation assessment is highly relevant in MDS with del(5q) and our data further suggest to closely monitor *TP53* mutated MDS patients with del(5q) with respect to clinical decision making due to their high risk of disease progression and low sensitivity to lenalidomide treatment [13]. Beyond previously published data on the influence of *TP53* gene mutations in MDS-iso5q [4], our data suggest two separate routes to CK with del(5q) in MDS. The main route includes CK cases arising from clones with a *TP53* mutation as the primary abnormality acquiring del(5q) later. Both haploinsufficiency of various genes in del(5q) and the TP53 pathway are exploited for (pre-)clinical drug development [14, 15]. Our data suggest considering the different routes of clonal evolution to CK in clinical testing of these drugs in MDS patients with del(5q) and *TP53* alterations. Although further research is necessary, it is tempting to speculate that novel drugs targeting the founder clone might be more effective than those targeting a subclonal event.

The comparison of different assays (FISH and NGS) for detection of del(5q) and *TP53* aberrations is a potential methodological limitation of our study. Although detection limits and precision metrics were well comparable in our hands, a systematic bias between these methods cannot be excluded. However, such a bias would influence both groups of patients equally. In addition, conclusions were only drawn from scenarios with sizable VAF differences and confirmed with more stringent cut-offs. Other limitations include the lack of follow-up information in several cases and missing data regarding CN-LOH of *TP53* due to the retrospective design. Thus, prospective studies are needed to consequently monitor genetic changes (clone size and/or gain of aberrations) in patients with isolated del(5q) in real-time in order to identify patients at risk (e.g., with *TP53* mutations) as early as possible.

In conclusion, clonal hierarchy analysis and serial genetic assessment suggest two separate routes to complex karyotypes with del(5q) in MDS: (1) deriving from MDS-iso5q which mainly harbor del(5q) within the ancestral clone and acquire *TP53* alterations during the course of the disease, (2) more commonly arise from clones with a *TP53* mutation as the primary abnormality acquiring del(5q) later.

## Supplementary information

Supplemental Material

## Data Availability

The datasets generated during and/or analyzed during the current study are available from the corresponding author on reasonable request.
