# Supplementary material for: Two ways to complex karyotype in MDS—the role of del(5q) and TP53
Source: Blood Cancer J. 2025 May 19;15(1):96. doi: 10.1038/s41408-025-01305-w (PMC12089310; doi:10.1038/s41408-025-01305-w)
Supplement: Supplementary file 1 — Supplemental Material [file 41408_2025_1305_MOESM1_ESM.docx]

**Supplementary Material**

**Supplementary Methods**

***Patients cohort and samples***

All MDS samples were sent to the MLL Munich Leukemia Laboratory between 01/2006 and 10/2023. Diagnoses (from peripheral blood and bone marrow) were made based on cytomorphology, cytogenetics and molecular genetics as previously published (1-3). To exclude other causes of cytopenia, additional immunophenotyping (3) was carried out if necessary. Samples used in this study were selected from a large cohort comprising 2,824 MDS cases. The inclusion criteria for this large cohort were i) a final diagnosis of MDS based on WHO classification valid at time of sampling, ii) cytomorphology analysis performed, iii) chromosome banding analysis (CBA) available, iii) molecular analysis of 23 myeloid genes (see below) and iv) fluorescence in situ hybridization (FISH) analysis of del(5q). All samples were further retrospectively reclassified following WHO-HAEM5 (Suppl. Table S1). For all patients the presence of cytogenetic abnormalities and mutations were obtained from the same sample. Follow-up samples were available in 150/729 patients. For progression analysis only patients with a minimum follow-up time of 90 days were considered.

***FISH analysis***

In order to determine the clone size of del(5q), FISH analysis was performed as previously described (1) using probes for the chromosomal region of 5q (XL Del(5)(q31) and/or XL Del(5)(q33); MetaSystems). Per sample 100 interphase nuclei were evaluated. The analytical sensitivity of the assay was validated as 5% positive nuclei (corresponding to a CA-VAF of 2.5%) and the precision as 4.99% coefficient of variation.

***Mutational analysis***

Mutational data was retrieved during routine work-up using targeted panel next generation sequencing (4) (median coverage 1500x). In detail, we evaluated 23 genes associated with myeloid malignancies using a variant allele frequency (VAF) cut-off of ≥3%: *ASXL1, BCOR, CBL, DNMT3A, ETV6, EZH2, FLT3, GATA2, IDH1, IDH2, JAK2, KIT, KRAS, MPL, NPM1, NRAS, RUNX1, SF3B1, SRSF2, TET2, TP53, U2AF1, ZRSR2*. Precision of the assay was validated as 3.91% coefficient of variation. *TP53* multi-hit was defined as either ≥2 *TP53* mutations, one *TP53* mutation with VAF >55% (indicating copy-neutral loss-of-heterozygosity, CN-LOH) or one *TP53* mutation accompanied by del(17p) (5).

***Clonal hierarchy analysis***

For hierarchy analysis VAFs of *TP53* were adjusted for overlaps with del(17p) (assessed by CBA/FISH) and overlaps with putative regions of CN-LOH (inferred by VAFs >55%) for them to be used as unbiased estimators for clone size; i.e., VAFs should correspond to half of the clonal proportion in the sample as for mutations in regular diploid backgrounds. For del(17p) VAFs were recalculated as VAF_adj_ = VAF / (1 + VAF). This makes the simplifying assumption that both the *TP53* mutation and del(17p) exclusively reside in the same clone. For putative CN-LOH VAFs were recalculated as VAF_adj_ = VAF / 2.

A cut-off of at least a 5% difference between (CA-)VAF estimates were used to evaluate clonal hierarchy of *TP53* and del(5q) (6, 7).

***Statistical analysis***

For statistical analyses R version 4.2.2 (R Foundation for Statistical Computing, Vienna, Austria) with the survival and survminer packages and SPSS version 19.0 (IBM Corporation, Armonk, NY) were used. Analyses for overall survival (OS) were performed according to Kaplan-Meier and compared using two-sided log rank tests. The OS was calculated as time from diagnosis to death or last follow-up. Dichotomous variables were compared using Fishers exact or chi-square test. For comparison of median values, the two-sided Mann-Whitney U test was applied. All results were considered significant at *p*<0.05.

**Supplementary Results**

***Hierarchy analysis with more stringent primary event definitions***

Given that VAF estimates for del(5q) and *TP53* stemmed from different methods and adjustments of *TP53* VAFs had to be performed in case of overlaps with del(17p) or CN-LOH we repeated our hierarchy analysis in two subsets of samples to investigate if our observed patterns were affected by any potential biases or uncertainties. Firstly, we used an increased VAF difference of ≥10% to define the primary event (Suppl. Figure S2A). Here the differences in between MDS subtypes remained visible and highly significant (*p*<0.001). Secondly, we performed the analysis on the subset of samples for which FISH showed a diploid genotype at the *TP53* locus and which concurrently displayed *TP53* VAFs ≤55% i.e., which were less likely to be affected by CN-LOH (MDS-iso5q: n=23, MDS-CK n=87). Here we compared unadjusted *TP53* VAFs to del(5q) CA-VAFs using a cut-off of ≥5% (Suppl. Figure S2B). Again, we confirmed a highly significant difference between entities (*p*<0.001).

***Detailed follow-up analysis***

From 84 MDS-iso5q patients data on karyotype and *TP53* status was available from different time points. Of those, 78 either developed CK (n=20) or showed a stable karyotype (n=58). Lenalidomide treatment was documented for 36 patients. Of those patients progressing to CK, patients with lenalidomide treatment (n=10) showed a median progression time to CK of 4 years [0.8-7.3] and patients without lenalidomide treatment (n=6) progressed within 1.1 years [0.5-5.3] (*p*=0.083). In 4 patients treatment information was missing. Of the 10 lenalidomide treated patients progressing to CK, one patient progressed from *TP53* single to multi-hit mutations while from 9 patients with wild-type *TP53* 5 progressed to multi-hit mutations. In 26 lenaliomide treated patients retaining their iso5q karyotype at follow-up, 4 had *TP53* mutations of those only one progressed to multi-hit and 22 were *TP53* wild-type of those 11 gained *TP53* mutations. Overall, from 36 lenalidomide treated patients 1/5 *TP53* mutated patients and 9/31 *TP53* wild-type patients progressed to CK. In addition, 2/5 *TP53* mutated and 16/31 *TP53* wild-type patients gained *TP53* mutations. There were no statistical differences regarding clinical outcome/ karyotype progression in patients treated with lenalidomide as samples sizes were too small.

Out of the 20 cases acquiring CK during progression, 13 harbored *TP53* alterations (10/13 multi-hit, 3 single-hit) in CK phase (Suppl. Figure S3B) while seven of these showed *TP53* alterations already in the MDS-iso5q phase (median VAF: 21%; 2 multi-hit, 5 single-hit). Interestingly, of the 13 MDS-iso5q patients lacking *TP53* mutations in MDS-iso5q phase and progressing to MDS-CK, 6 developed multi-hit *TP53* alterations (5/6: lenalidomide treatment) and 7 retained their *TP53* wild-type status (3/7: lenalidomide treatment). Our data indicate that in a subset of MDS-iso5q patients *TP53* alterations are gained during progression from MDS-iso5q to MDS-CK leading to either single-hit *TP53* mutated MDS-CK cases starting from TP53 wildtype MDS-iso5q or leading to multi-hit *TP53* mutated MDS-CK cases starting from single mutated *TP53* MDS-iso5q.

**Supplementary Tables and Figures**

**Table S1. Cohort overview**

| Characteristics | All del(5q) cases | MDS-iso5q | MDS-CK | “other” |
| --- | --- | --- | --- | --- |
| **Number of samples** | 729 | 308 (42%) | 365 (50%) | 56 (8%) |
| **Age** (years; median [range]) | 76 [32-98] | 77 [35-91] | 74 [32-98] | 78 [37-87] |
| **Sex** (female / male) | 58% / 42% | 72% / 28% | 46% / 54% | 55% / 45% |
| **Bone marrow blast count** (%; median [range]) | 4.5 [0-19.5] | 4 [0-19] | 7 [0-19.5] | 4.5 [0-19.5] |
| **Mutations per sample** (n; median [range]) | 1 [0-6] | 1 [0-5] | 1 [0-5] | 1 [0-6] |
| **Cases with mutations** (n; %) | 614 (84%) | 226 (73%) | 341 (93%) | 47 (84%) |
| **WHO-HAEM4R** |  |  |  |  |
| MDS-del(5q) | 235 (32%) | 212 (68%) | 0 (0%) | 12 (41%) |
| MDS-SLD/MLD  +/-RS | 87 (12%) | 0 (0%) | 84 (23%) | 3 (5.4%) |
| MDS-EB-1 | 146 (20%) | 48 (16%) | 84 (23%) | 14 (25%) |
| MDS-EB-2 | 154 (21%) | 30 (10%) | 116 (32%) | 8 (14.3%) |
| t-MDS | 91 (13%) | 11 (4%) | 72 (20%) | 8 (14.3%) |
| Morphology not  evaluable | 16 (2%) | 7 (2%) | 9 (2%) | 0 (0%) |
| **WHO-HAEM5** |  |  |  |  |
| MDS-5q | 230 (32%) | 209 (68%) | 0 (0%) | 21 (37%) |
| MDS-bi*TP53* | 286 (39%) | 16 (5%) | 259 (71%) | 11 (20%) |
| MDS-LB | 27 (4%) | 0 (0%) | 25 (7%) | 2 (4%) |
| MDS-IB1 | 93 (13%) | 46 (15%) | 34 (9%) | 13 (23%) |
| MDS-IB2 | 76 (10%) | 30 (10%) | 38 (10%) | 8 (14%) |
| AML | 2 (<1%) | 0 (0%) | 1 (<1%) | 1 (2%) |
| Morphology not  evaluable | 15 (2%) | 7 (2%) | 8 (2%) | 0 (0%) |
| pCT | 91 (13%) | 11 (4%) | 72 (20%) | 8 (14%) |
| **Clinical data** |  |  |  |  |
| Follow up sample - availability | 150 (21%) |  |  |  |
| Survival data  - availability |  | 168 (55%) | 182 (50%) |  |

“other”: non-isolated del(5q), non-complex karyotype, also includes del(5q) in independent clone.

**
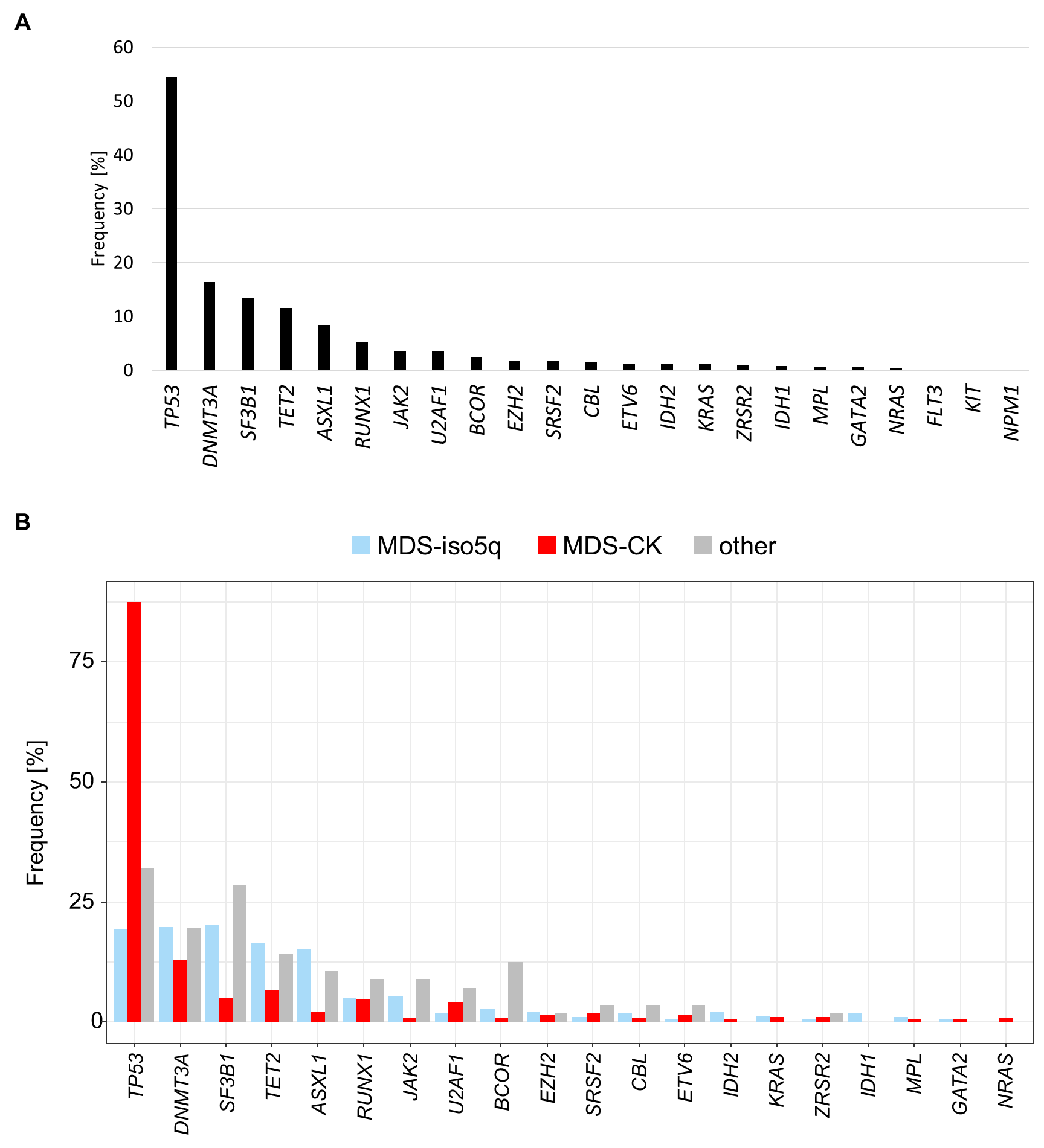
**

**Supplementary Figure S1: Co-mutation pattern of MDS with del(5q).** Co-mutations of all 729 cases MDS with del(5q) **(A)** and separated by subgroups **(B)**. MDS-iso5q: n=308; MDS-CK: n=365; other: n=56; CK: complex karyotype.

**
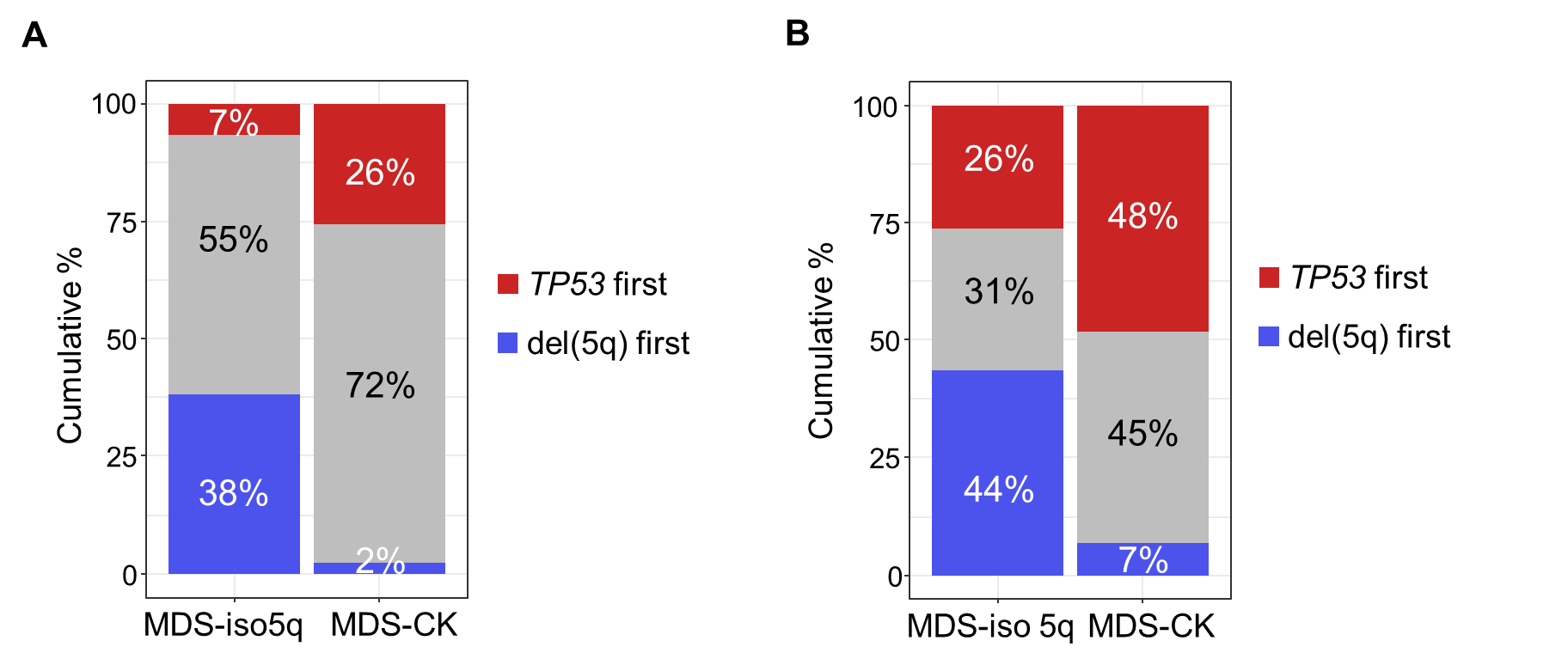
**

**Supplementary Figure S2: Primary events using more stringent definitions.** Cumulative percentage of *TP53* mutations or del(5q) as primary event separated by entity. **(A)** Using an increased the VAF difference cut-off to ≥10%. **(B)** Only including samples with diploid genotype at the *TP53* locus and presumed lack of CN-LOH.


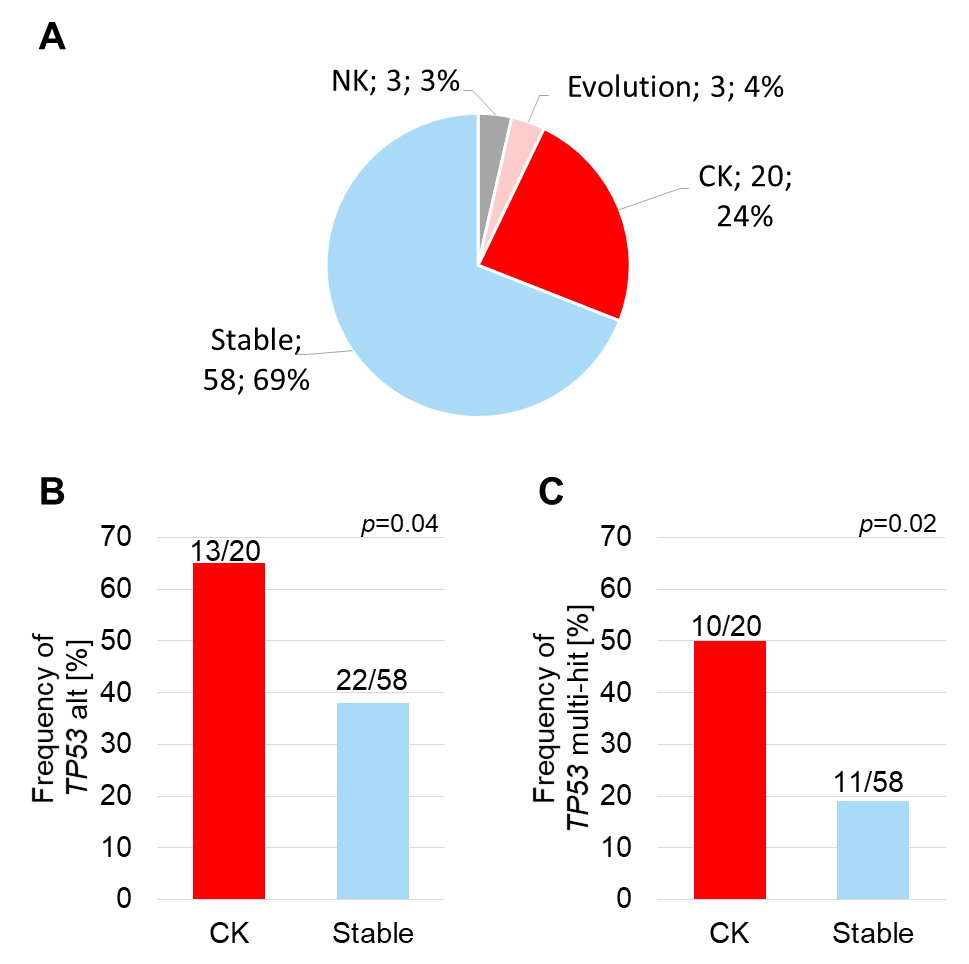


**Supplementary Figure S3: Follow-up analysis of 84 MDS-iso5q patients. (A)** Distribution of patients with normal karyotype (NK), clonal evolution, CK or stable karyotype at final follow-up. Absolute numbers of patients are shown. CK: complex karyotype. Frequencies of *TP53* alt **(B)** and of multi-hit *TP53* alt **(C)** within patients progressing to CK (red) and those retaining a stable karyotype (blue). CK: complex karyotype; alt: alterations.

**
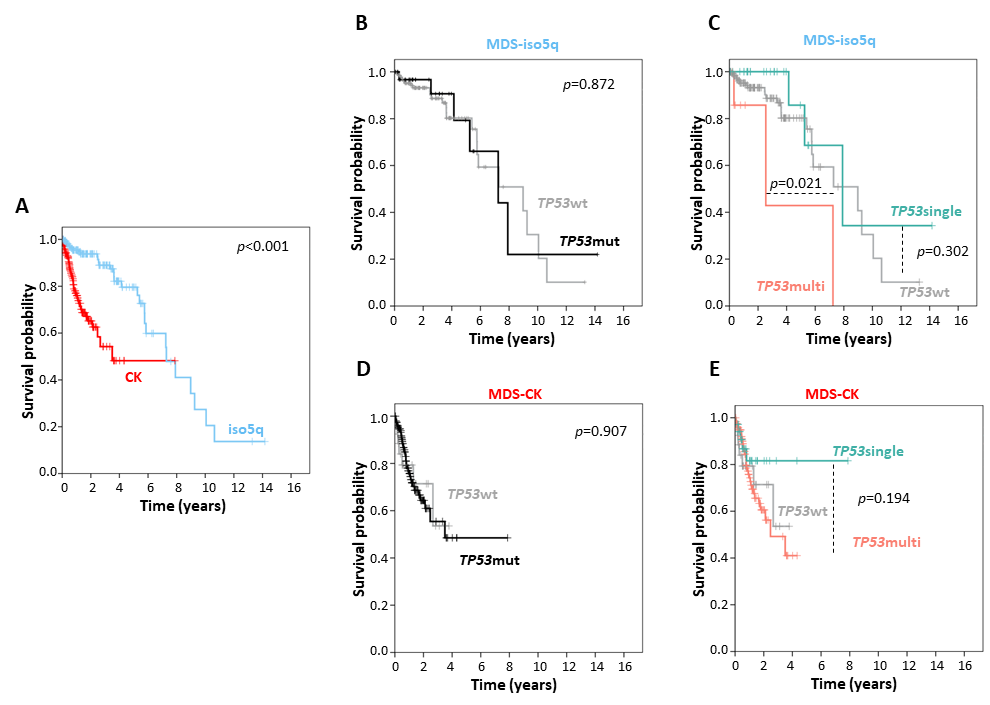
**

**Supplementary Figure S4: Survival analyses of MDS-iso5q and MDS-CK. (A)** Overall survival (OS) analysis of MDS patients with iso5q (n=168; median: 7.3 years) compared to CK (n=182; median: 3.5 years). **(B)** OS analysis of MDS patients with iso5q (n=168) comparing patients with *TP53* mutations (mut; n=33; median: 7.2 years) to patients with wild-type *TP53* (wt; n=135; median: 9.0 years). **(C)** OS analysis of MDS patients with iso5q (n=168) stratified for *TP53* allele status (single-hit: n=25 with median 7.9 years OS; multi-hit: n=8 with median 2.5 years OS). *TP53*multi vs. *TP53*single: *p*=0.021; *TP53*single vs. *TP53*wt: *p*=0.302. **(D)** OS analysis of MDS-CK patients (n=182) comparing patients with *TP53* mutations (mut; n=155; median: 3.5 years) to patients with wild-type *TP53* (wt; n=27; median OS: not reached). **(E)** OS analysis of MDS-CK patients (n=182) stratified for *TP53* allele status (single-hit: n=35 with median OS not reached; multi-hit: n=120 with median 2.5 years OS). *TP53*multi vs. *TP53*single: *p*=0.194.

## References

1. Schoch C, Schnittger S, Bursch S, Gerstner D, Hochhaus A, Berger U, et al. Comparison of chromosome banding analysis, interphase- and hypermetaphase-FISH, qualitative and quantitative PCR for diagnosis and for follow-up in chronic myeloid leukemia: a study on 350 cases. Leukemia. 2002;16(1):53-9.

2. Haferlach T, Kern W, Schoch C, Hiddemann W, Sauerland MC. Morphologic dysplasia in acute myeloid leukemia: importance of granulocytic dysplasia. J Clin Oncol. 2003;21(15):3004-5.

3. Kern W, Voskova D, Schoch C, Hiddemann W, Schnittger S, Haferlach T. Determination of relapse risk based on assessment of minimal residual disease during complete remission by multiparameter flow cytometry in unselected patients with acute myeloid leukemia. Blood. 2004;104(10):3078-85.

4. Maierhofer A, Mehta N, Chisholm RA, Hutter S, Baer C, Nadarajah N, et al. The clinical and genomic landscape of patients with DDX41 variants identified during diagnostic sequencing. Blood Adv. 2023; doi:10.1182/bloodadvances.2023011389.

5. Grob T, Al Hinai ASA, Sanders MA, Kavelaars FG, Rijken M, Gradowska PL, et al. Molecular characterization of mutant TP53 acute myeloid leukemia and high-risk myelodysplastic syndrome. Blood. 2022;139(15):2347-54.

6. Hirsch CM, Nazha A, Kneen K, Abazeed ME, Meggendorfer M, Przychodzen BP, et al. Consequences of mutant TET2 on clonality and subclonal hierarchy. Leukemia. 2018;32(8):1751-61.

7. Palomo L, Meggendorfer M, Hutter S, Twardziok S, Adema V, Fuhrmann I, et al. Molecular landscape and clonal architecture of adult myelodysplastic/myeloproliferative neoplasms. Blood. 2020;136(16):1851-62.
